# Flame Retardancy Evolution Behavior and Molecular Mechanism of Polyvinyl Chloride Under the Action of Damp Heat Aging

**DOI:** 10.3390/polym17060794

**Published:** 2025-03-17

**Authors:** Ke Xu, Chenyu Gao, Xin Liu, Xiuzhen Liu, Ganxin Jie, Jun Deng, Xinhan Qiao, Wentian Zeng

**Affiliations:** 1 State Key Laboratory of Environmental Adaptability for Industrial Products, Guangdong Provincial Key Laboratory of Environmental Adaptability Technology for New Energy Products and Materials, China National Electric Apparatus Research Institute Co., Ltd., Guangzhou 510663, China; xuk@cei1958.com (K.X.); xinliu@cei1958.com (X.L.); xiuzhenliu@cei1958.com (X.L.); ganxinjie@cei1958.com (G.J.); 2 State Grid Xuzhou Power Supply Company, Xuzhou 221000, China; gcy0731@126.com; 3 Electric Power Research Institute, Ultrahigh Voltage Transmission Company, China Southern Power Grid Co., Ltd., Guangzhou 510663, China; 4 School of Electrical Engineering, China University of Mining and Technology, Xuzhou 221116, China; qiaoxinhan@cumt.edu.cn (X.Q.); ts23230005a31@cumt.edu.cn (W.Z.)

**Keywords:** flame retardancy, polyvinyl chloride, molecular mechanism, damp heat aging, cable insulation

## Abstract

A 56-day aging test of polyvinyl chloride (PVC) cable material under hot and humid conditions was conducted, followed by tests on flame retardancy after varying degrees of wet heat aging, including vertical burning behavior, oxygen index, and afterflame time. Using molecular dynamics simulation theory, the molecular mechanism behind the changes in flame-retardant properties after wet heat aging was investigated based on experimental observations. The results indicate that, as wet heat aging progresses, the flame brightness decreases, the oxygen index increases, and afterflame and afterglow times significantly decrease in vertical combustion tests. These findings suggest that the flame-retardant properties of PVC improve as moist heat aging deepens. After aging, the combustibles within PVC samples diffuse more easily, and the precipitation of CaCO_3_ on the PVC surface enhances surface density, intermolecular forces, and thermal stability, which are key factors in the improved flame retardant performance.

## 1. Introduction

Polyvinyl chloride (PVC) is an electrically insulating thermoplastic polymer and is widely used due to its excellent heat resistance [1,2]. PVC is currently the primary material used in low-voltage cables [3,4], where it forms the outer sheath in combination with fillers like CaCO_3_ [5,6]. However, like most polymers, PVC is flammable, posing a combustion risk when exposed to internal short-circuit overloads or external heat sources during cable operation [7]. Studies indicate that cable fires are one of the leading causes of cable failures [8]. In environments such as chemical plants and thermal power stations, cable fires can trigger chemical explosions or fires in oil-immersed transformers, leading to significant equipment damage and losses in life and production [9]. Thus, investigating the flame-retardant properties of PVC is essential to ensuring the safe and reliable operation of cables.

Researchers have extensively explored combustion characteristics of PVC cables. Fernandez-Pello et al. conducted an experimental study on ignition delay times and upward flame spread rates on the insulated cable surface under external radiation flux [10]. Xie et al. examined the fire resistance of new and old PVC cables using thermogravimetric (TG) and Fourier transform infrared (FTIR) methods, revealing that older cables burned more thoroughly [8]. Further studies on the pyrolysis and flammability of PVC cable sheathing showed that older cables were less flammable than newer ones [6,11]. An et al. investigated the flammability and flame spread characteristics of PVC cables, analyzing flame shape, size, spread speed, and temperature distribution [9]. Yan et al. explored the thermal, flammability, and degradation behavior of cable sheaths as well as their aging mechanism in salt spray and hygrothermal environments [12]. With the increasing demand for environmental protection, phosphorus flame retardants and inorganic flame retardants have been widely used. Researchers have also carried out many studies on flame retardants. For example, Artem et al. synthesized amino acid rifosphazines and tested their effect as a flame retardant for epoxy resin [13]. Terekhov et al. studied flame retardancy of an epoxy resin composition based on resin d.e.r.-330, isomethyltetrahydroxythalic anhydride and new epoxy-containing aryoxyclotriphosphazenes [14]. Vahabi et al. discussed the influence of environmental aging on the fire response behavior of flame retardant polymers and summarized six factors that can change the fire resistance of materials [15]. However, during operation, cables are exposed to various stresses, such as electrical and thermal stresses, which can cause insulation materials to age over time. This aging degrades both insulation and flame-retardant properties, significantly increasing fire risks for power equipment [16,17]. The changes in flame-retardant properties of PVC before and after aging are therefore critical concerns. To ensure the safe and stable operation of PVC cables throughout their lifespan, it is essential to study the long-term flame-retardant performance of insulating materials under the combined influence of working conditions.

At present, research on materials such as cable insulation layers and outer sheaths primarily focuses on the aging characteristics under the influence of a single factor and the effects of aging on mechanical and insulation properties. Zhang et al. conducted accelerated thermal aging tests on cables and examined changes in the mechanical properties and frequency domain dielectric spectra of cable materials at different aging stages [18]. Liu et al. carried out thermal aging tests on cross-linked polyethylene cables under gamma radiation, using gas chromatography and nuclear magnetic resonance to reveal the aging mechanism [19]. Additionally, studies have explored the changes in the physicochemical properties of high-voltage cable materials under UV thermal aging [20]. Bibo Wang et al. investigated the flame-retardant property changes in high-impact polystyrene after natural aging in Turpan and Huaibei, China, concluding that the synergistic effects of different environmental conditions caused varying degrees of polymer chain breakage, flame-retardant migration, and material erosion, leading to differences in flame-retardant performance [21]. These studies mainly focus on the aging behavior of high-voltage cable materials under oxygen, temperature, and radiation conditions. However, there are few reports on the aging process of PVC low-voltage cable materials and how this aging affects their flame-retardant properties. In southern China and countries near the equator, due to frequent rainy seasons, cables operate in environments primarily influenced by humidity and temperature. Based on this analysis, investigating the aging behavior of PVC cables under the combined effects of humidity and temperature, as well as the impact of aging on their flame-retardant properties, is essential to ensuring the safe operation of low-voltage cables in high-humidity areas.

In recent years, the study of microscopic mechanisms based on macroscopic experimental phenomena of insulating materials has become a hot topic in current research [22,23,24]. Using molecular simulation technology, researchers have made significant progress in exploring the microscopic mechanisms of insulating material aging, changes in electrical properties, and other related processes [25,26,27]. Applying molecular simulation to reveal the mechanisms behind the changes in flame-retardant properties of PVC materials during wet heat aging holds important theoretical value [28,29]. It provides insights into assessing fire hazards and predicting the lifespan of PVC materials, ensuring their safe application in environments with high humidity.

In this paper, an aging test of PVC cable material was conducted under hot and humid conditions for 56 days. Further tests on flame retardancy of PVC with varying degrees of wet heat aging were performed, including vertical burning phenomena, oxygen index measurements, and afterflame time assessments. Finally, using molecular dynamics simulation theory, the molecular mechanism underlying the changes in flame-retardant properties of PVC after wet heat aging was investigated in relation to the experimental findings. This study provides both experimental data and theoretical support for the safe operation of PVC low-voltage cables in humid regions.

## 2. Experimental and Simulation Methods

### 2.1. PVC Wet Heat Aging Test

According to the ISO 294 standard [30], PVC test samples were prepared by compression molding, with CaCO_3_ filler included in the samples. The dimensions of the PVC strip specimens were as follows: length 125 ± 5 mm, width 13.0 ± 0.5 mm, and thickness 3.0 ± 0.1 mm. A total of no fewer than 70 strip specimens were prepared and divided into 7 groups (S0~S6), with at least 10 specimens in each group. The humidity and heat aging test of the PVC samples was conducted using the KFH-1000SF1A0 high- and low-temperature humidity test chamber, produced by Guangdong Keming Environmental Instrument Industry Co., Ltd., Guangdong, China. Following the IEC 60068-2-78:2012 standard [31], the PVC samples underwent wet heat aging testing. The environmental conditions were maintained at a temperature of 85 ± 5 °C and a relative humidity of 90 ± 10%. Samples S0–S6 correspond to samples aged for 0, 1, 4, 10, 21, 35, and 56 days, respectively.

During the test, the initial temperature is set at 20 °C and then increased to 85 °C within 2 h. Humidity parameters are set via the electronic control screen, and through gradient settings, the experimental environment is maintained at approximately 90% humidity. The aging time for each sample is then maintained according to the wet and hot conditions specified, and the experimental device is closed to complete the process. After the wet and hot aging test, the samples are pre-treated at 23 ± 2 °C and 50 ± 5% relative humidity for at least 48 h.

### 2.2. Test Method for Flame-Retardant Properties of PVC

According to the IEC 60695-11-10 standard [32], the horizontal combustion test is conducted to measure the linear burning rate of PVC samples under specified conditions. The test involves clamping one end of the sample firmly and positioning it horizontally. The free end of the sample is then exposed to a test flame with its height adjusted to 20 mm. The flame is applied to the sample for 30 s or continuously until the flame reaches the 25 mm mark on the sample. The burning time required for the flame to travel from the 25 mm mark to the 100 mm mark is recorded. This test assesses the flammability and combustion behavior of the material under controlled conditions.

According to the IEC 60695-11-10 standard [32], the vertical combustion test measures the self-extinguishing ability of materials under specified conditions. In this test, one end of the PVC strip sample is held horizontally, while the free end is exposed to a test flame with a height of 20 mm. The flame is applied to the sample for 10 s, and the afterflame time t1 is recorded. The flame is then reapplied for another 10 s, and both the afterflame time t2 and afterburning time t3 are recorded. Alternatively, the flame is applied continuously until it reaches the 25 mm mark. The burning time required for the flame to travel from the 25 mm mark to the 100 mm mark is also recorded. This test assesses the material’s ability to self-extinguish and its burning behavior under vertical conditions.

Ultimate oxygen index determination test: oxygen index refers to the minimum oxygen concentration required to maintain the combustion of a material when a mixture of oxygen and nitrogen at a temperature of 23 °C ± 2 °C is introduced under specific conditions. During the measurement, the sample is fixed vertically in a transparent combustion tube filled with an upward-flowing mixture of oxygen and nitrogen. The top of the sample is then ignited, and a series of tests under different oxygen concentrations are conducted by comparing the continuous combustion time or combustion length of the sample so as to estimate the minimum oxygen concentration required to maintain combustion.

According to the ISO 22309 standard [33], the microstructure of PVC samples was analyzed. The microstructure of PVC was scanned using a TESCAN MIRA scanning electron microscope, produced by Tescan in Brno, Czech Republic. The types and contents of elements in PVC samples were analyzed using a QUANTAX electrically cooled spectrometer, produced by Bruker in Karlsruher, Germany.

### 2.3. Mechanism of Change in Flame-Retardant Properties of PVC Based on Molecular Dynamics Simulation

The mechanism analysis of the change in flame-retardant properties of PVC was carried out from two perspectives: the overflow of combustible gas in PVC and the precipitation of CaCO_3_ on the surface. The internal structure model of unaged PVC containing CaCO_3_ was constructed, and the degree of polymerization of PVC was set to 10. With the continuous development of wet heat aging, the molecular chain of PVC broke, and the internal CaCO_3_ gradually decreased and precipitated to the surface. Based on the unaged model, the PVC was short-chained, and the content of CaCO_3_ was reduced, and the internal structure model of PVC after aging was constructed. The same amounts of combustible gases C_2_H_2_ and C_2_H_4_ were added into the two models, respectively [34].

Based on this model, the COMPASS force field was used to carry out molecular dynamics simulation of combustible gas in the PVC model. First, to ensure the model’s accuracy, geometry optimization, annealing, and model dynamic equilibrium were required. All models were optimized with 10,000 steps. Secondly, a 1 ns model equilibrium simulation was carried out based on the NPT ensemble (constant-pressure and constant-temperature ensemble) to achieve a reasonable density value. Then, a 1 ns simulation was carried out based on the NVT ensemble (canonical ensemble with constant molecular number, volume, and temperature). Finally, the diffusion behavior and differences in combustibles in the unaged and aged PVC models were analyzed based on the trajectories obtained from the NVT simulation. The periodic boundary condition was used in the whole simulation process. A Nose thermostat and Berendsen barostat were adopted during the simulation, while the Verlet equation of motion was integrated using a time step of 1 fs. The simulation temperature was set to 85 °C.

**Figure 1:**
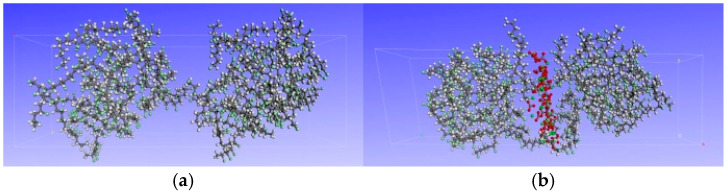
PVC surface molecular model before and after aging: (**a**) unaged PVC surface model, (**b**) aged PVC surface model.

According to the test results, CaCO_3_ gradually precipitates on the surface of PVC after aging (see Section 4.1 for detailed results). This is of great significance to the improvement of flame-retardant properties of PVC. Therefore, models with and without CaCO_3_ on the surface of PVC were constructed, as shown in Figure 1. The simulation process and parameters are basically the same as above. The simulation temperature was set to the combustion temperatures of PVC: 140 °C, 240 °C, 340 °C, and 440 °C.

## 3. Effect of Wet Heat Aging on Combustion Characteristics of PVC

### 3.1. PVC Vertical Burning Phenomenon During Wet Heat Aging Process

**Figure 2:**
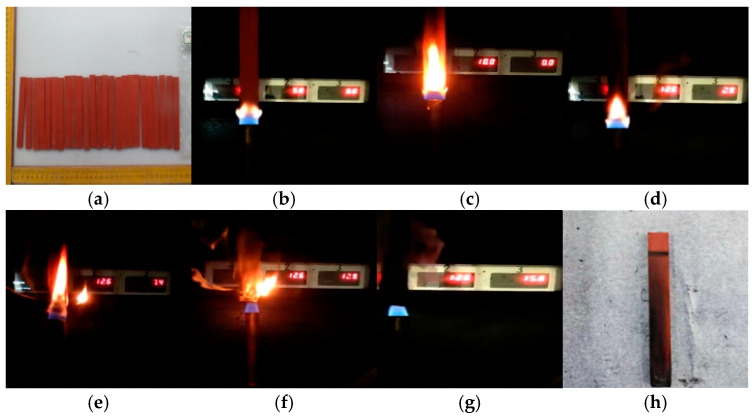
Changes in the vertical burning phenomenon after PVC aging for 4 days: (**a**) before combustion, (**b**) 5 s, (**c**) 10 s, (**d**) 15 s, (**e**) 20 s, (**f**) 25 s, (**g**) 28 s, (**h**) after burning.

Taking the PVC sample aged for 4 days as an example, the changes in the combustion phenomenon of the PVC sample over time are compared, as shown in Figure 2. The color and size of the PVC samples did not change significantly after aging. During the combustion process, some areas of the sample appeared black, and the residual material after combustion remained quite complete and compact, with no dripping matter. In addition, some massive raised solids were also observed on the surface of the sample. It is worth noting that a clear flame was observed at the beginning of the combustion, but at 25 s the flame flickered and was accompanied by obvious smoke. However, as time went on, the flame gradually weakened and eventually extinguished itself. For the unburned part, its surface was covered with black smoke volatiles released during combustion. Given that PVC undergoes obvious melting decomposition during combustion, a large amount of gases such as HCl and water vapor are produced. HCl continues to burn after mixing with oxygen, while water vapor has a certain inhibitory effect on the combustion process. Therefore, it is inferred that this interaction may be the cause of the flame flickering.

**Figure 3:**
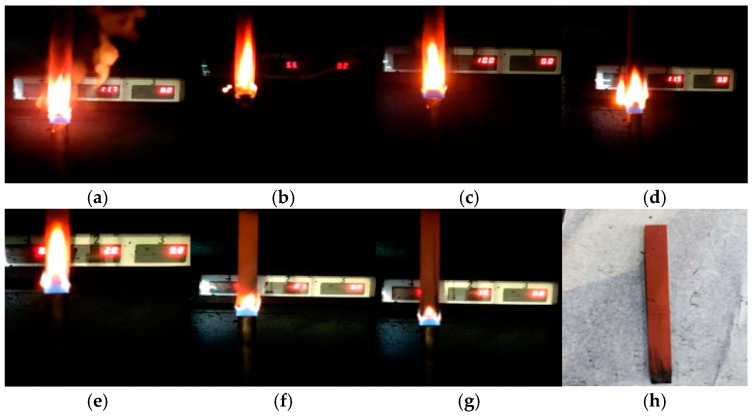
Vertical combustion phenomenon of PVC at different aging times: (**a**) S0, (**b**) S1, (**c**) S2, (**d**) S3, (**e**) S4, (**f**) S5, (**g**) S6, (**h**) sample of S6 after combustion.

Figure 3 compares the vertical burning phenomenon of PVC samples with different aging times. When the PVC materials in groups S0–S6 were first ignited, the flames were bright, and flickering flames were observed in all these groups. However, as the aging time progressed, the combustion phenomenon changed significantly. The flames gradually became less bright, and the color and density of the smoke also showed a decreasing trend. Overall, the combustion of PVC is suppressed as the aging time increases.

PVC materials produce thick smoke during combustion and extinguishing, mainly due to the incomplete combustion of carbonaceous substances. Therefore, in areas where PVC materials are put into use, it is recommended to install smoke extraction facilities and smoke suppression equipment to effectively mitigate the potential harm of smoke to the human body to ensure the safety and health of personnel.

### 3.2. Change of Oxygen Index of PVC Combustion During Wet Heat Aging Process

**Figure 4:**
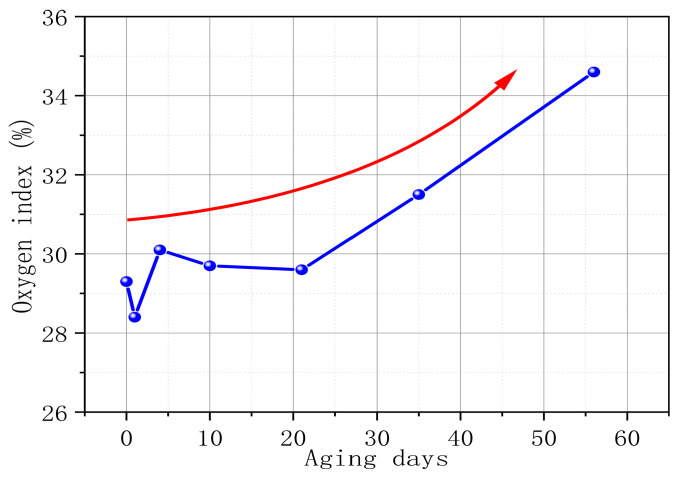
PVC oxygen index changes with aging time.

The oxygen index refers to the minimum oxygen concentration required for a material to burn with a flame in an oxygen–nitrogen mixed gas flow under specified conditions. It is expressed as the volume percentage of oxygen. A high oxygen index means that the material is not easy to burn, and a low oxygen index means that the material is easy to burn. Figure 4 tests the change in PVC oxygen index with aging time. As the aging time increases, the oxygen index of PVC shows an obvious growth trend. From S0 to S6, the oxygen index of PVC increases by 18.09%. This phenomenon further supports our view point that the higher the degree of PVC aging, the less likely it is to burn. In addition, it is worth noting that the oxygen index of PVC in the first three stages of the aging process fluctuates. This phenomenon is mainly due to the precipitation of internal additives (such as antioxidants, plasticizers, and additives) and the diffusion of gas products in the early stage of PVC aging.

### 3.3. Changes in Afterflame and Afterglow Time of PVC During Wet Heat Aging Process

**Figure 5:**
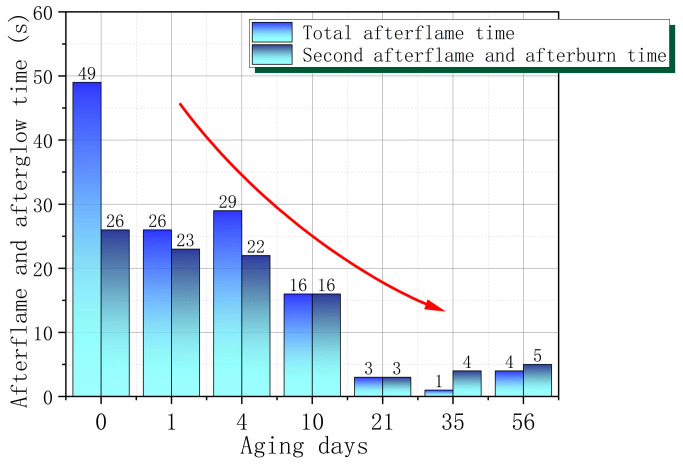
PVC afterflame and afterglow time changes with aging time.

Figure 5 shows the afterflame and afterglow time during the vertical combustion test of PVC with different aging times. It can be observed that the overall second afterflame and afterglow times of PVC decrease with increasing aging time, especially after 21 days, the second afterflame and afterglow times decrease significantly. In addition, from the S0 to S7 stage, the combustion flame gradually becomes weaker and the self-extinguishing speed is faster. The oxygen index change data further confirm that the flame-retardant performance of PVC improves as the aging time increases, and after 21 days, the flame retardant performance is significantly improved. This means that the ignition risk of PVC samples after wet heat aging is relatively low. In other words, once the newly used PVC material is ignited, the danger of its burning spreading and helping the fire to cause a disaster is greater. Therefore, for the newly used PVC material, special attention must be paid to prevent ignition from external fire sources or strong heat sources.

### 3.4. Fire Rating of PVC Samples

**Figure 6:**
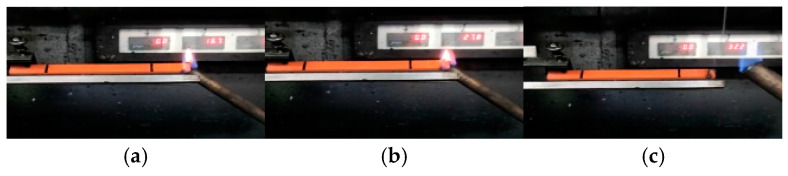
S1 group PVC horizontal burning phenomenon: (**a**) mid-stage combustion phenomenon, (**b**) post-stage combustion phenomenon, (**c**) samples after combustion.

The horizontal burning test is an important method for evaluating the fire resistance level of materials. Considering the reference significance of the vertical burning test and oxygen index combustion data, it is more representative to select aged materials with relatively weak flame-retardant properties for horizontal burning analysis. Figure 6 records the horizontal combustion test process of the S1 group PVC samples. The test results show that after ignition, the PVC samples did not produce dripping. After extinguishing, their burning length did not exceed the 25 mm mark. Based on these performances, it is determined that the fire protection level of these materials is HB40.

## 4. Molecular Mechanism of Improved Flame-Retardant Properties of PVC After Wet Heat Aging

### 4.1. Microstructure Changes in PVC Before and After Wet Heat Aging

**Figure 7:**
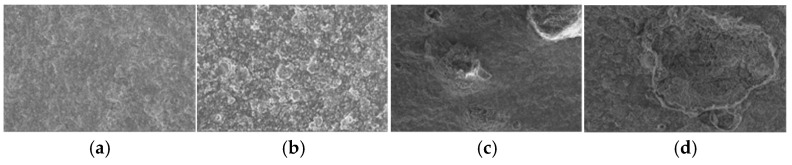
Microstructure of PVC samples: (**a**) S0-1000×, (**b**) S0-6000×, (**c**) S6-1000×, (**d**) S6-6000×.

The surface microstructure of PVC samples was analyzed before aging (S0) and after 56 days of aging (S6) using scanning electron microscopy, as illustrated in Figure 7. The microscopy images reveal that the surface of the S0 group is notably smoother and more refined compared to the S6 group. In contrast, the S6 group exhibits a greater number of raised structures on the surface. These raised structures are essentially channels formed due to the precipitation of fillers from within the material during the aging process.

**Figure 8:**
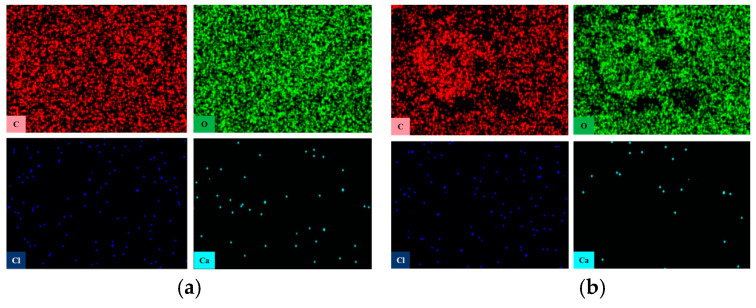
Element distribution of PVC samples: (**a**) S0, (**b**) S6.

Furthermore, the element distribution of PVC samples was analyzed using an energy dispersive spectrometer, as illustrated in Figure 8. Table 1 details the changes in the primary elements on the surface of the PVC samples. With the increase in aging time, the carbon content on the surface of the PVC samples shows a slight decrease, while the O and Ca content exhibit a significant rise. This observation supports the hypothesis that CaCO_3_ gradually migrates from the interior of the material to the surface. Since the combustion of PVC begins at the surface, the increased CaCO_3_ concentration on the surface makes the material less flammable. This phenomenon is a key factor in the enhanced flame retardant performance of PVC after wet heat aging.

### 4.2. Diffusion Behavior of Combustibles in PVC Samples Before and After Aging

**Figure 9:**
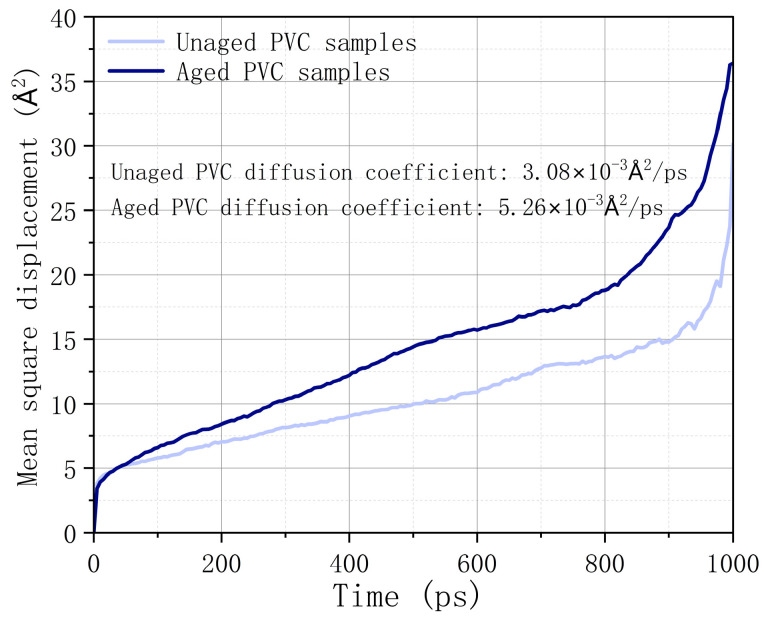
Mean square displacement of combustibles in different PVC models.

Figure 9 presents the calculated mean square displacement and self-diffusion coefficient of combustibles in different PVC samples. The results show that as the simulation time progresses, the diffusion capacity of combustibles in unaged PVC samples is lower compared to that in aged PVC samples. Specifically, the self-diffusion coefficient of combustibles in aged PVC samples is approximately 1.71 times higher than in unaged PVC samples. This indicates that as the aging process advances, combustibles are more likely to escape from the aged PVC sample, which in turn reduces the risk of combustion. This finding supports the conclusion that the flame-retardant properties of PVC improve with aging due to the facilitated outflow of combustibles.

**Figure 10:**
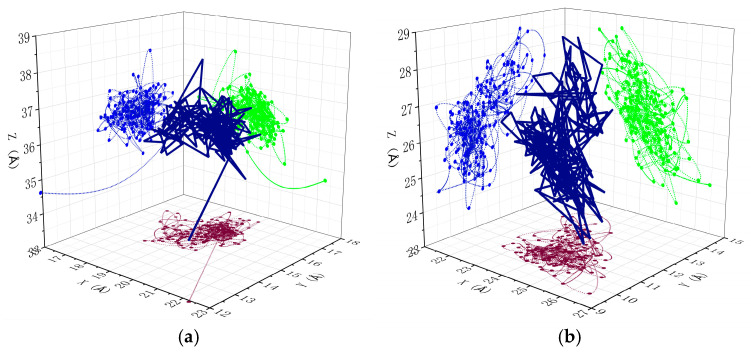
Diffusion trajectory of combustibles in different PVC models: (**a**) unaged PVC sample, (**b**) aged PVC sample.

Figure 10 displays the diffusion trajectories of combustibles in unaged and aged PVC samples, providing further confirmation of the aforementioned conclusions. The figure shows the diffusion trajectory of combustibles in PVC samples and their projected trajectories on the X, Y, and Z axes. For consistency in comparison, the scales of the XYZ axes were standardized for both unaged and aged PVC samples. The simulation results indicate that combustibles in unaged PVC exhibit a more compact diffusion pattern, with a relatively smaller diffusion range across all three spatial directions (X, Y, and Z). In contrast, as the aging degree of the PVC samples increases, the diffusion range of combustibles expands and becomes more dispersed, particularly in the Y and Z directions. This observation reinforces the idea that aging enhances the mobility and diffusion of combustibles, contributing to the improved flame retardant performance of PVC over time.

The interaction energy between combustibles and PVC samples plays a critical role in determining the differences in diffusion behavior observed in unaged and aged PVC samples. As shown in Table 2, the interaction energy primarily consists of van der Waals forces, with a minor contribution from Coulombic interactions. A comparison of the interaction energies before and after aging reveals that the structural changes in aged PVC reduce the interaction energy between the combustibles and the PVC matrix. This decrease in interaction energy in aged PVC samples is the key factor facilitating the easier diffusion and overflow of combustibles. Consequently, this reduced interaction allows combustibles to move more freely within the material, which leads to improved flame-retardant properties as aging progresses.

### 4.3. Thermal Stability of PVC Surface Before and After Aging

**Figure 11:**
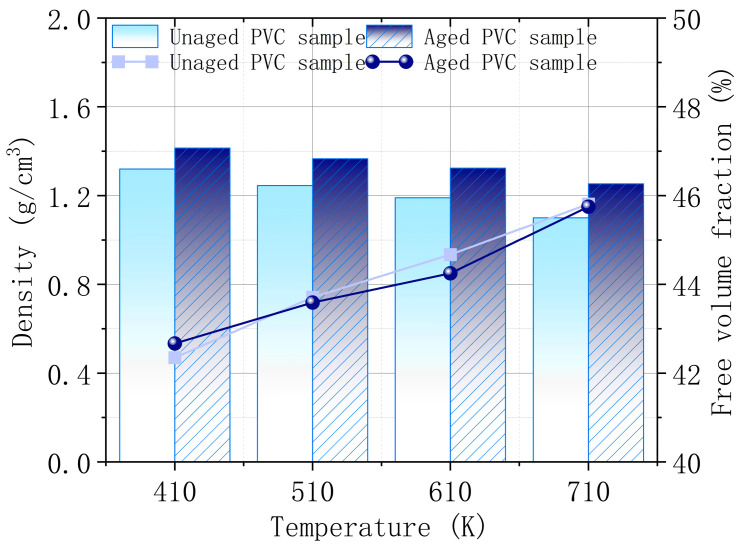
Density and free volume fraction of PVC surface before and after aging.

The calculation results in Figure 11 indicate that the density of the PVC surface increases, and the free volume fraction decreases as CaCO_3_ precipitates during aging. This increased surface density enhances the thermal stability of the material, while the reduced free volume fraction suggests fewer voids between molecules, limiting molecular mobility. The greater density and reduced free volume mean that the PVC surface becomes more compact, resulting in stronger intermolecular forces and better thermal stability. At four typical combustion temperatures, the aged PVC samples consistently exhibit improved thermal stability compared to the unaged ones. However, as the temperature rises, both the density and free volume fraction of PVC samples show a decreasing and increasing trend, respectively. This indicates that the higher the temperature, the more molecular expansion occurs, weakening the material’s thermal stability and making it more prone to combustion. These findings align with the observed behavior of PVC during the combustion tests, where higher temperatures make PVC more flammable, particularly in the unaged samples.

**Figure 12:**
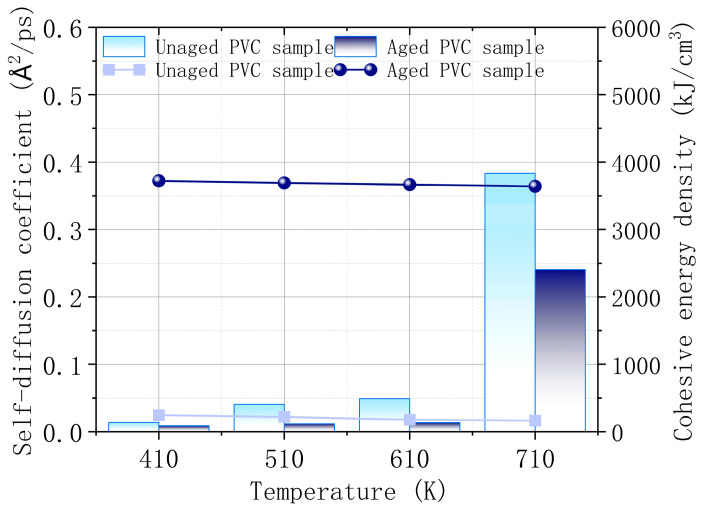
Self-diffusion coefficient and cohesive energy density of PVC surface before and after aging.

Figure 12 illustrates the changes in the self-diffusion coefficient and cohesive energy density of the PVC surface before and after aging at different temperatures. The results indicate that the self-diffusion coefficient of the PVC surface is higher before aging compared to after aging, across all temperature ranges. This suggests that molecular mobility is greater in unaged PVC, which may facilitate the diffusion of combustibles and promote combustion. As the temperature rises, the self-diffusion coefficient increases significantly, indicating a marked decline in thermal stability at higher temperatures, regardless of the aging status. The cohesive energy density, which reflects the strength of intermolecular forces, shows a clear increase in aged PVC samples. This is attributed to the precipitation of CaCO_3_ on the surface, which enhances the intermolecular forces, thereby improving the thermal stability of the PVC material. Stronger intermolecular forces reduce the likelihood of combustion reactions, making aged PVC more resistant to burning. Thus, the simulation confirms that the CaCO_3_ precipitate not only restricts the movement of molecules but also plays a crucial role in enhancing the flame-retardant properties of PVC after wet heat aging.

## 5. Conclusions

In this paper, the changes in the flame-retardant properties of PVC samples after hygrothermal aging were systematically studied. The molecular mechanisms underlying the alterations in flame-retardant properties of PVC before and after hygrothermal aging were uncovered. The main conclusions of this paper are as follows:

(1) As the moist heat aging time of PVC samples increases, the flame brightness diminishes, the oxygen index rises, and the afterflame and afterglow times significantly shorten during the vertical combustion test. These observations suggest that the flame-retardant properties of PVC samples improve as the moist heat aging process progresses. Additionally, the horizontal burning test results classify the fire rating of the PVC samples as HB40.
(2) The volatilization of combustible substances inside PVC samples during wet heat aging is a key factor in enhancing their flame-retardant properties. After wet heat aging, typical combustible substances exhibit stronger diffusion abilities and wider diffusion trajectories within the aged PVC samples. This is primarily due to the reduced interaction between the aged PVC samples and the combustibles compared to unaged PVC samples, allowing the combustibles to diffuse more easily and thereby reducing the risk of combustion.
(3) During the wet heat aging process, the precipitation of CaCO_3_ on the surface of PVC plays a crucial role in enhancing its flame-retardant properties. This CaCO_3_ precipitation increases the surface density, cohesive energy density, and intermolecular forces, while simultaneously reducing the free volume fraction and self-diffusion coefficient. As a result, the thermal stability of the aged PVC surface becomes significantly stronger. At typical combustion temperatures ranging from 410 K to 710 K, the aged PVC surface exhibits better thermal stability compared to the unaged PVC surface, further improving its resistance to combustion.

## 6. Prospect

This study investigates the effects of damp heat aging on the combustion performance of PVC materials from a macroscopic perspective by conducting combustion tests, oxygen index tests, and other experiments on samples subjected to varying durations of damp heat aging. Subsequently, the damp heat aging process of PVC materials is simulated using molecular modeling techniques, providing a microscopic explanation for the changes in combustion performance. To fully elucidate the underlying mechanisms, further studies, including smoke density tests and pyrolysis analysis, are required. Nevertheless, this research offers a novel perspective on the changes in flame retardancy of PVC materials due to aging, providing a new and feasible direction for subsequent studies.

## Figures and Tables

**Table 1:** Changes in main elements on the surface of PVC samples.

Element	S0	S6
C	56.04	40.47
O	39.13	50.18
Cl	2.96	1.75
Ca	1.87	7.6

**Table 2:** Interaction energy between combustibles and different PVC samples (kcal/mol).

	Unaged PVC Smaple	Aged PVC Smaple
Interaction energy	−123.77	−118.99
Vdw interaction energy	−97.33	−89.26
Coulomb interaction energy	−26.44	−29.73

## Data Availability

Data are contained within the article.
